# Hot-Melt Processed Glibenclamide Glassy Solutions: A Novel Oral Delivery Platform for Enhanced Bioavailability in Diabetes

**DOI:** 10.3390/pharmaceutics18040421

**Published:** 2026-03-30

**Authors:** Hany S. M. Ali, Ahmed F. Hanafy, Ahmed Almotairy, Marey Almaghrabi, Hamad Alrbyawi, Waleed A. Mohammed-Saeid

**Affiliations:** 1 Department of Pharmaceutics and Pharmaceutical Industries, College of Pharmacy, Taibah University, Madinah 42353, Saudi Arabia; 2 Health and Life Research Center, Taibah University, Madinah 42353, Saudi Arabia; 3 Research and Development Department, Al-Andalous Pharmaceutical Industries, Giza 12573, Egypt; drafathy@gmail.com

**Keywords:** hot-melt injection molding, glibenclamide, glassy solutions, miscibility, oral bioavailability

## Abstract

**Background/Objectives**: Hot-melt injection molding (HMIM) was evaluated as a solvent-free process for the preparation of glibenclamide (GLB), a poorly soluble BCS Class II drug, glassy solutions with the objective of improving dissolution and bioavailability for diabetes. **Methods**: GLB was blended at a concentration of 10% *w*/*w* with PVP K25, PVP VA64, and Soluplus^®^ (SOL) matrices. The miscibility of the GLB–polymer systems (matrices) was calculated based on the Hansen solubility parameters and validated using differential scanning calorimetry (DSC) analysis. The HMIM extrudates were milled into granules and analysed for their solid-state properties (DSC, XRPD, FTIR, and SEM studies), and flow properties. The produced granules were compressed into immediate release tablets and assessed for in vitro performance, stability, and in vivo bioavailability using 20 healthy male Sprague Dawley rats. **Results**: Findings revealed the formation of single-phase glassy solutions, specifically for PVP VA64 and SOL, which also exhibited advantageous manufacturing and extrudate clarity. The glassy solution formulations showed considerably improved dissolution characteristics compared with the crystalline GLB and the commercial product. The glassy solution formulations displayed fast drug release for PVP K25 and PVP VA64, and biphasic drug release for SOL. Stability testing confirmed the capability of PVP VA64 and SOL to maintain GLB in a molecularly dispersed, amorphous state for 12 months. The in vivo assessment revealed an increase in relative bioavailability to 246.3% and 124.5% for the SOL and PVP VA64 formulations when compared to the commercial formulation. **Conclusions**: Overall, the findings demonstrate the potential of HMIM-processed glassy solutions, especially those prepared using SOL, as promising platforms for promoting oral delivery of the poorly soluble antidiabetic GLB.

## 1. Introduction

One of the critical issues associated with class II BCS compounds is their limited oral bioavailability, despite efficient permeability [1]. Such drugs require improvement in dissolution rates in the biological environment, especially in the gastrointestinal system [2]. The technique of solid dispersion is widely adopted to address the dissolution rate-related limitation of such drugs. In solid dispersions, the drug substance is dispersed in a polymeric carrier, resulting in the formation of an amorphous, high-energy state with apparently higher solubility [3]. Among these, molecular dispersions (glassy solutions) in which the drug substance is dispersed homogeneously on a molecular level within a polymeric carrier, exhibit better physical stability and dissolution rate enhancement characteristics relative to the traditional amorphized form of dispersion systems [4,5].

In this respect, hot-melt injection molding (HMIM) has been recognized as an emerging solvent-free and continuous process in the pharmaceutical industry. HMIM involves firstly melting the drugs and the polymers in a heated chamber. The mixed fluid is subsequently injected into a mold cavity using high pressure. The fluid solidifies when cooled in the mold cavity to form a predetermined three-dimensional structure [6]. This technique presents several clear advantages when preparing solid dispersions and final dosage forms. HMIM is fully solvent-free; issues related to residual solvents, time-consuming drying procedures, and environmental concerns are thus avoided. Furthermore, HMIM enables the manufacturing of highly reproducible final products with precise dimensional and weight control [7]. These advantages make HMIM particularly suitable for preparing molecularly dispersed systems of poorly soluble drugs.

Glibenclamide (GLB), a commonly used sulfonylurea in type 2 diabetes mellitus, is a BCS Class II drug that is less soluble yet more permeable [8]. The aqueous solubility of GLB is approximately 0.04 mg/mL with a log P (octanol-water partition coefficient) value of 3.53, and pKa of 4.32 [9,10]. The drug exhibits pH-dependent solubility, with higher solubility in alkaline media (approximately 3.67 ± 0.012 µg/mL at pH 7.4) and lower solubility in acidic gastric conditions (2.13 ± 0.05 µg/mL at pH 1.2) [11]. Its pharmacological potency is exclusively hindered by oral absorption variability. Therefore, developing GLB as a glassy solution could be considered a promising alternative to realize its high bioavailability. Choosing a suitable carrier material to support such a solution plays a crucial role in developing a successful formulation. Conventional carriers such as polyvinylpyrrolidone (PVP K25) are traditionally proven, while more advanced carriers such as Soluplus^®^ (SOL) or (PVP VA64, Kollidon VA64), are considered to possess special properties [12]. Soluplus^®^, which has been shown to be amphipathic, has been reported to improve solubility, suppress precipitation, or both, via forming a protective micellar matrix, while PVP VA64 has been shown to exhibit remarkable miscibility with high processability with poorly soluble compounds [13,14,15].

This study aims to examine the suitability of hot-melt injection molding (HMIM) as an integrated manufacturing platform for developing immediate-release GLB tablets with enhanced drug dissolution and bioavailability. Specifically, the study evaluated (i) drug–polymer miscibility and solid-state properties; (ii) preparation of GLB glassy solution granules via HMIM; (iii) compression into immediate-release tablets; and (iv) in vitro stability and in vivo performance evaluation in comparison to commercially produced GLB tablets.

## 2. Methodology

### 2.1. Materials

The European Egyptian Pharmaceutical Industries (Pharco Corporation, Alexandria, Egypt) kindly gifted glibenclamide (GLB with a median particle size of 4.84 μm). BASF (Ludwigshafen, Germany) supplied PVP K25 (Kollidone K25^®^), Soluplus^®^ (polyvinyl caprolactam-polyvinyl acetate-polyethylene glycol graft co-polymer, and SOL) and Kollidon^®^VA64 (vinylpyrrolidone-vinyl acetate copolymers (PVP VA64)). Pharmaceutical grades of other ingredients were used.

### 2.2. Assessment of GLB Solubility in Tested Polymers

#### 2.2.1. Miscibility of GLB–Polymers

The drug–polymer miscibility of GLB and PVP K25, PVP VA64 or SOL was predicted using the Hildebrand solubility parameter (δ), and the Flory–Huggins interaction parameter (χ) [16]. This approach is widely applied for pre-formulation screening of drug–polymer compatibility in amorphous solid dispersions (ASDs) [17]. The Hansen solubility parameter (δt) used was 23.8 MPa^1/2^ for GLB [18], 22.2 MPa^1/2^ for PVP K25 [19], 23.7 MPa^1/2^ for PVP VA64 and 22.1 MPa^1/2^ for Soluplus [20]. These values were obtained from the dispersion (δ_d_), polar (δ_p_), and hydrogen-bonding (δ_h_) contributions according to Equation (1):

(1) $$\delta_{t}=\sqrt{\delta_{d}^{2}+\delta_{p}^{2}+\delta_{h}^{2}}$$

The difference in solubility parameters (Δδ) between GLB and each polymer was computed using Equation (2) [21]:

*Δδ* = ∣*δ_GLB_* − *δ_Polymer_*∣ (2)

The widely accepted miscibility criterion was as follows: Δδ values < 7.0 MPa^1/2^ point to a high probability of miscibility, while Δδ values > 10.0 MPa^1/2^ indicate immiscibility. The Δδ values computed were interpreted to classify each polymer as miscible, partially miscible, or immiscible with GLB.

The difference in total solubility parameters between GLB and each polymer (Δδ) was then used to estimate χ based on the following Equation (3):

(3) $$\chi =\frac{V_{d}}{RT}(\delta_{GLB}-\delta_{polymer}{)}^{2}$$

where $V_{d}$ is the molar volume of glibenclamide (cm^3^·mol^−1^), R is the universal gas constant (8.314 J·mol^−1^·K^−1^), and T is the absolute temperature (298 K). Lower χ values indicate stronger drug–polymer affinity and reduced phase separation tendency, whereas χ > 0.5 generally reflects limited miscibility and an increased tendency toward phase separation [22].

#### 2.2.2. DSC Analysis

The thermal properties of GLB and the individual polymers (PVP K25, PVP VA64, and SOL), GLB–polymer extrudates and physical mixtures were measured were measured using the DSC technique (Netzsch F3 Maia^®^ thermal analyzer, Selb, Germany). For analysis, perforated aluminium pans containing specific weights of the tested samples (3–4 mg) were used. The thermal protocol consisted of two heating cycles: samples were first heated from 0 to 200 °C at a scanning rate of 10 °C/min, then cooled to 0 °C (20 °C/min), followed by a second heating cycle to 200 °C at the same rate. Nitrogen served as a purge gas at a flow rate of 50 mL/min to maintain an inert atmosphere [17,23]. The glass transition temperature (T_g_) was determined from the second heating cycle of DSC thermograms as the midpoint (T_g_-mid) of the heat capacity step using the instrument software (Proteus^®^ Version 6.1.0, Netzsch, Selb, Germany). All T_g_ determinations were conducted with consistent use of the same analysis parameters. The change in heat capacity (ΔC_p_) was determined by measuring the vertical step height between the extrapolated baselines of the glassy and the supercooled liquid states in a DSC curve.

#### 2.2.3. T_g_ Prediction

The Gordon–Taylor Equation (4) predicted the T_g_ of GLB–polymer binary mixtures.

(4) $$T_{g}=\frac{W_{1\;}T_{g1}+K\;W_{2}T_{g2}}{W_{2}+KW_{2}}$$

where T_g x_ is the glass transition temperature of each component, Wx represents weight fractions of the components, and subscript 2 represents the compound with the higher T_g_ value. K in the previous equation was estimated from the densities ($\rho_{x}$) and T_g_ of the amorphous components of GLB, PVP K25, SOL, and PVP VA64, respectively, using the Simha–Boyer Equation (5), which relates K to the ratio of densities with glass-transition temperatures of the pure components [23,24,25]:

(5) $$k=\frac{\rho_{1}T_{g1}}{\rho_{2}T_{g2}}$$

where ρ_1_ and T_g1_ represent the respective values of the density and the glass transition temperature of the amorphous component with the lowest T_g_, whereas ρ_2_ and T_g2_ represent the corresponding values of the density and glass transition temperature of the amorphous component with the highest T_g_. The Simha–Boyer rule assumes that the change in the thermal expansion coefficient at T_g_ is similar for many amorphous materials, allowing k to be approximated from density and T_g_ data alone [2,3,4]. True densities of amorphous components were collected from former publications as 1.37 for GLB [18], 1.18 for PVP K25 [26], 0.99 for SOL [20] and 1.167 g/cm^3^ for PVP VA64 [20]. The T_g_ values of the pure materials were 62 °C for GLB [27], 145 °C for PVP K25 [26], 101 °C for PVP VA64 and 71 °C for SOL [20]. A concentration of 10% GLB in the solid solution granules was selected for this T_g_ prediction testing. The 10% *w*/*w* GLB loading was considered to be optimal for three reasons. First, the DSC screening indicated complete amorphization of GLB at this ratio across all polymers. Second, tablet manufacturing requirements necessitated this loading to achieve the target 2.5 mg dose in a 200 mg tablet that matches the reference marketed product for comparative in vitro dissolution and bioavailability testing. Third, physical stability considerations dictated that lower drug loading enhances anti-plasticization and molecular separation, thereby ensuring robust stability [28]. The predicted T_g_ values for the drug–polymer mixtures obtained from the Gordon–Taylor equation were compared with experimentally determined T_g_ values. Closeness between the predicted and experimental T_g_ values indicates efficient glassy solution formation and can be considered evidence of homogeneous single-phase formation at the molecular level [23].

### 2.3. Preparation of GLB Glassy Solution Extrudates

Formulation mixtures of GLB with each polymer (PVP K25, SOL, or PVP VA64) at a concentration of 10% *w*/*w* were prepared by sequential dilution using a mortar and pestle (i.e., initially mixing a small quantity of GLB with an equal quantity of polymer, then progressively adding the polymer in increments while continuously mixing to ensure homogeneous distribution). Each prepared mixture was loaded into the melting cylinder, heated to a specified temperature. A piston was then installed to compress the powder and allow air removal for 15 min until a clear melt was obtained. The melted mixture was injection molded using a benchtop piston injection molding system (HAAKE™ MiniJet Pro Piston Injection Molding System, Thermo Scientific, Waltham, MA, USA). The cylinder temperature was set to 150 °C for the 10% GLB-PVP VA64 and 10% GLB-SOL formulations, and to 180 °C for the 10% GLB-PVP K25 formulation. The mold temperature was maintained at 20 °C. The injection molding process consisted of two stages: first, an injection pressure of 800 bar for 40 s, followed by a post-pressure of 150 bar for 10 s. The melt was injected through a 1 mm nozzle into a cylindrical mold cavity (0.5 cm in width and 1 cm in depth). The mold cavity was pre-lubricated with vegetable oil every 15 injections to facilitate extrudate extraction. After molding, the mold was held at 20 °C for 1 min to allow the extrudate to cool and harden before extraction. The obtained extrudates were visually evaluated for clarity on a scale of 1 to 3 (1: clear, 2: slightly hazy, and 3: unclear). Process manufacturability was considered successful if a complete extrudate was retrieved; it was deemed unsuccessful or of low efficiency if the extrudate was broken or liquefied. Solid solution granules were prepared from the extrudates using a cutter-type grinder (Microfine grinder drive MF 10 Basic, IKA, Switzerland) at 3000 rpm with 0.25 mm and 0.5 mm inserts. Granule fractions were then sieved using 500 µm standard sieves.

### 2.4. Flowability Evaluation

The resulting glassy solution granules (GSGs) from each polymeric formula (GLB-PVP K25, GLB-PVP VA64, and GLB-SOL) were then tested for the powdered flow properties, which are critical for downstream tablet manufacturing. The bulk density was determined in terms of pouring a known weight of granules (about 10 g) into a graduated cylinder without any tapping, and the volume was measured. The tapped density was determined using an automatic tap density machine (Pharma Test, Hainburg, Germany) employing the United States Pharmacopoeia <616> guidelines. The density machine tapped the cylinder 500 times with the volume being measured when no further change in volume was observed. Carr’s index (CI) or compressibility index was determined using Equation (6), given below, for flowability:

(6) $$\mathrm{Carr}’s\;\mathrm{Index}\;(\%)=\frac{\rho_{t}-\rho_{b}}{\rho_{t}}\times 100$$

where ρ t = tapped density, and ρ b = bulk density. Based on USP <1174> classification, flowability of the granules was found to be excellent (CI ≤ 10%), good (11–15%), fair (16–20%), passable (21–25%), poor (26–31%), very poor (32–37%), and extremely poor (> 38%). All experiments were conducted in triplicates, and data were expressed in mean ± standard deviation.

### 2.5. Tableting

In this study, milling and tableting were used to allow standard pharmaceutical tableting evaluation, including compaction properties and tablet mechanical strength. Direct molded tablets could produce dense polymer matrices that could hinder rapid drug release, especially with high polymer content. The compositions of the solid solution loaded tablet formulations are presented in Table 1. The glassy solution granules (GSGs) sieved through a 500 µm mesh were mixed with microcrystalline cellulose (MCC), Crospovidone, and colloidal silicon dioxide for 5 min in a glass beaker fitted with an impeller (Ika mixer, Switzerland) set to 500 rpm. Magnesium stearate was then added and mixed at the same speed for an additional 3 min. An 8 mm punch on a single-punch machine (Erweka, Langen, Germany) was used to compress each formulation of the GSG mixtures into tablets. The weight of the tablet was set at 200 mg, and the compression force (8–10 kN) was selected to achieve a friability of less than 0.5% while maintaining a disintegration time close to that of the reference product, at less than 1 min. The solid fraction of the resulting tablets was calculated as 0.754, 0.755, and 0.774 for GLB-PVP K25-tab, GLB-PVP VA64-tab, and GLB-SOL-tab, respectively. All tablets contained 12.5% glassy solution granules, which themselves contained 10% GLB.

### 2.6. Tablet Characterization

Tablet hardness was measured with an Erweka (Heusentamm, Germany) hardness tester (*n* = 10). A Pharma Test friability tester (Hainburg, Germany) was used in determination of the percentage weight loss of tablets, where a sample of ten tablets was subjected to 300 rotations, and the percentage weight loss was calculated. Disintegration of GLB tablets was recorded after observing six tablets until they were fully disintegrated in water at 37 °C. GLB content was evaluated by crushing four randomly selected tablets. GLB was extracted from 200 mg of the resulting powder using methanol. After filtering the suspension, the GLB content was quantified using an HPLC assay (as detailed in the Section 2.13). This analysis was performed on three different samples, with results expressed as an average with a standard deviation [29].

### 2.7. X-Ray Powder Diffraction (XRPD)

The crystallinity of GLB within milled extrudate granules (<0.5 mm) was also evaluated by X-ray powder diffraction (XRPD). Measurements were performed on a Shimadzu XRD 6000 diffractometer (Shimadzu Corporation, Kyoto, Japan) with a scanning range (2θ) from 10° to 60°. The system utilized a copper radiation source (λ = 1.5418 Å) and a graphite monochromator at a speed of 0.04°/minute.

### 2.8. Fourier-Transform Infrared Spectroscopy (FTIR)

The infrared spectra of unprocessed GLB, PVP K25, SOL, PVP VA64, and their corresponding combinations were collected by FT-IR spectroscopy. The analysis was conducted on an IRAffinity-1 spectrophotometer (Shimadzu, Japan) using attenuated total reflectance (ATR) mode, with scans from 4000 to 750 cm^−1^.

### 2.9. Scanning Electron Microscopy (SEM)

The SEM technique was used to examine the morphological features of GLB solid solution granules. Micrographs were taken using a scanning electron microscope (JEOL Ltd., Tokyo, Japan) operating at 10 kV. The granules were fixed to specimen stubs with double-sided carbon tape and sputter-coated with gold using a plasma sputter coater (GSL-1100X-SPC-12, MTI Corporation, Shenyang, China). Samples were visualized at about 270–300X magnification.

### 2.10. In Vitro Dissolution Testing

The in vitro dissolution profiles of unprocessed GLB, tablet formulations (GLB-PVP K25-tab, GLB-PVP VA64-tab, GLB-PVP VA64-tab, and GLB-SOL-tab) and a commercial reference product were characterized by USP apparatus 2 (paddle type; Erweka, Germany). Dissolution tests were conducted in 900 mL (phosphate buffer, pH 6.8) and maintained at 37 °C, and the paddle speed was set to 75 rpm, as a commonly used discriminating medium. The rationale for selection of dissolution conditions was based on several factors. GLB is an acidic drug (pKa 4.32), exhibiting pH-dependent solubility with minimal dissolution at gastric pH and enhanced solubility in intestinal pH environments [11]. The pH of 6.8 provides acceptable discriminatory power between tested formulations while avoiding the poor solubility at gastric pH that would preclude drug release [8,30]. The 75 rpm agitation simulates in vivo hydrodynamics without masking formulation differences [31], while the 900 mL volume (standard USP conditions) ensures the sink conditions of GLB dissolution [32,33]. Aliquots of 5 mL were withdrawn at specific intervals (10, 20, 30, 45, 60, and 120 min) and immediately replaced with fresh medium. After filtration through a 0.2 µm filter (Millipore, Ireland), the GLB content in the samples was quantified via HPLC assay (as detailed in the Section 2.13). Drug release profiles were evaluated using the similarity factor (*f*_2_). This factor represents the “closeness” of two dissolution curves. The f2 factor can be calculated by Equation (7) [34]:

(7) $$f_{2}=50\cdot log\left\{{\left[1+\frac{1}{n}\sum_{t=1}^{n}(R_{t}-T_{t}{)}^{2}\right]}^{-0.5}\cdot 100\right\}$$

where $R_{t}$ and $T_{t}$ represent the cumulative percentage of drug dissolved at each time point t for the reference and test formulations, respectively. As per the FDA guidance, two dissolution profiles are counted as similar or equivalent when the calculated f2 value is >50.

### 2.11. Dissolution Kinetic Analysis

The dissolution profiles of the developed formulations (GLB-REF TAB, GLB-SOL TAB, GLB-PVP VA64 TAB, and GLB-PVP K25 TAB) were analysed using commonly applied mathematical kinetic models to elucidate the mechanism of drug release. The models applied included zero-order, first-order, Higuchi, and Hixson–Crowell models. The goodness of fit for each model was evaluated using the coefficient of determination (R^2^) obtained from linear regression analysis.

### 2.12. Stability Evaluation

A long term (25 °C, 60% RH) stability study was performed per ICH Q1A(R2) guidelines to confirm the physical stability of the glassy solid solutions and their tablets. Solid solutions and tablets were stored protected from light in high density polyethylene bottles for 12 months. DSC was employed to evaluate the effect of temperature and humidity on the physical stability of the solid solutions. Dissolution tests for glassy solution loaded tablets were re-conducted to assess stability.

### 2.13. Bioavailability

#### 2.13.1. Sampling

The Taibah University Ethics Committee pre-approved the research protocol including the research objectives, experimental design, methodology, and statistical analysis plan (COPTU-REC-83-20231123, on 23 November 2023), before the initiation of the in vivo studies. The study was not prospectively registered in a publicly accessible database. In vivo studies followed the standards outlined in the Guide for the Care and Use of Laboratory Animals, 8th edition (National Academies Press, USA). Experimental animals were procured from the animal house of Taibah University, Madinah, Saudi Arabia. The animals were of conventional health status and were genetically unmodified. This study employed 20 healthy male Sprague Dawley rats of conventional health status weighing 250 ± 20 g, split into four groups of five each. To avoid any confounding factors, animals from different treatment groups were kept under the same environmental conditions and handled by the same trained personnel. Before starting, rats were acclimatized in an animal facility with standard conditions such as 22 °C ± 3 °C, 50% ± 5% RH, and light/dark cycles of 12/12 h with food and water ad libitum for 5 days, with an overnight fast after that. For oral dosing, suspensions of tablets (in deionized water) were administered to rats using blunt intragastric tubing (10 mg GLB/kg). The commercial tablet was administered to Group I, while Groups II, III, and IV received the GLB-PVP K25, GLB-PVPVA 64, and GLB-SOL formulations, respectively. Blood samples were collected using heparinized tubes at standardized intervals of 0.25, 0.5, 1, 2, 4, 8, 12, and 24 h after administration. Centrifugation of the blood sample at 13,000 rpm for 10 min was done immediately after collection and samples were then stored until analysis. Every animal was observed for any signs of adverse events such as changes in behaviour, physical appearance or respiratory distress. During the 24-h research period, no adverse events were noted in any of the tested groups. All 20 rats lived to the last blood collection time point, and none of the animals achieved the humane endpoints (such as inability to reach food/water, persistent recumbency or convulsions) during the trial. Formulation dosing and blood sampling were done in a balanced manner to prevent any time bias.

#### 2.13.2. Processing and Quantification

All plasma samples were processed and quantified identically by a researcher who was blinded to the allocation of experimental groups. Each sample was treated with 0.5 mL of acetonitrile to precipitate the plasma proteins. After a minute of vortexing, the samples were centrifuged at 5000 rpm for five minutes. The supernatant was carefully transferred to a new Eppendorf tube, and 20 μL was injected into a validated HPLC system for quantification of GLB [35]. A Shimadzu Prominence HPLC System (Shimadzu, Kyoto, Japan) with a diode array detector (SPD-M20A) was used to conduct the analysis. The calibration curve, generated in rat plasma, spanned from 0.01 to 0.4 µg/mL with gliclazide as the internal standard. A mobile phase containing 0.05 M phosphate buffer (pH 3.5) and acetonitrile (40:60% *_V_*/*_V_*) was pumped isocratically at a flow rate of 1 mL/min after being filtered and degassed. The reverse-phase Hypersil™ BDS C18 column (4.6 mm × 150 mm, 5 μm; Thermo Fisher Scientific, Waltham, MA, USA) was used for separation. Ultraviolet absorbance was measured at 228 nm for detection.

#### 2.13.3. Pharmacokinetic Analysis

The values of C_max_ and T_max,_ matching the maximal plasma concentration and corresponding time were determined directly from the individual plasma concentrations of tested animals. Trapezoidal rules were applied to calculate the area under the curve from zero to 24 h (AUC_0–24h_). The relative ratio, [AUC_test_/AUC_reference_] denoted the percentage relative bioavailability (F%). One-way analysis of variance (ANOVA) was utilized to conduct statistical analysis of the obtained pharmacokinetic parameters (SPSS 16 software, SPSS Inc., Chicago, IL, USA). Pharmacokinetic parameters were presented as mean ± standard deviation (SD). A *p* value of less than 0.05 signified statistical significance.

## 3. Results and Discussion

### 3.1. Assessment of GLB Solubility with Polymers

It is important to select the right type of polymeric material to produce solid dispersion formulations by using the method of injection molding, especially when the active ingredient in the formulation is poorly soluble, as in the case of GLB. The polymeric carriers tested here were PVP K25, PVP VA64, and SOL. The rationale for selection was based on their distinct physicochemical properties and their demonstrated efficacy within amorphous solid dispersion (ASD) formulations. PVP K25, a homopolymer of vinylpyrrolidone, was chosen as a benchmark hydrophilic carrier, given its established capacity to generate solid dispersions and impede crystallization via hydrogen bonding interactions. Its comparatively elevated T_g_ (≈145 °C) offers effective anti-plasticizing effects, though it necessitates elevated processing temperatures [12]. PVP VA64, a vinylpyrrolidone-vinyl acetate copolymer (60:40), was chosen for its intermediate characteristics; the vinyl acetate component diminishes hygroscopicity relative to PVP homopolymers while preserving favourable drug–polymer miscibility [36]. The lower T_g_ (≈101 °C) of PVP VA 64, compared to PVP K25, facilitates processing at reduced temperatures [37]. SOL, a graft copolymer of polyvinyl caprolactam, polyvinyl acetate, and polyethylene glycol, was chosen as a sophisticated amphiphilic carrier, displaying surfactant characteristics. This amphiphilic architecture, characterized by a hydrophilic PEG backbone and hydrophobic vinyl caprolactam and vinyl acetate side chains, allows for solubilization via micelle formation and the prevention of precipitation. Moreover, the low T_g_ (≈71 °C) of SOL enables processing at temperatures notably lower than the melting point of GLB [38]. The capability of GLB to form an amorphous solid dispersion with these polymeric carriers was investigated using theoretical miscibility predictions, thermal analysis, and experimental validation of glass transition behaviour.

#### 3.1.1. Estimation of GLB–Polymer Miscibility

As shown in Table 2, the δ values for GLB, PVP K25, PVP VA64, and SOL were 23.8, 22.2, 23.7, and 22.1 MPa^1/2^, respectively. The respective estimated Δδ values were 1.6, 0.1, and 1.7 MPa^1/2^ for PVP K25, PVP VA64, and SOL. All Δδ values were below the threshold, 7.0 MPa^1/2^, which suggests a high probability of miscibility of GLB with each polymer. The Flory–Huggins interaction parameter is a thermodynamic descriptor of drug–polymer affinity and is frequently employed to rationalize miscibility, phase behaviour, and physical stability of ASDs [39]. Smaller χ values indicate stronger drug–polymer interactions, reduced Gibbs free energy of mixing, and a lower thermodynamic driving force for phase separation and crystallization [40]. All GLB–polymer systems exhibited χ values below 0.5, suggesting that homogeneous dispersions are thermodynamically feasible. Notably, the extremely low χ value observed for GLB–PVP VA64 (0.0015) implies near-ideal miscibility. These findings are consistent with previous works highlighting the significance of solubility parameters for effectively predicting the production of stable amorphous solid dispersions of BCS Class II drugs [12].

#### 3.1.2. DSC Characterization at Different Drug Loadings

**Figure 1:**
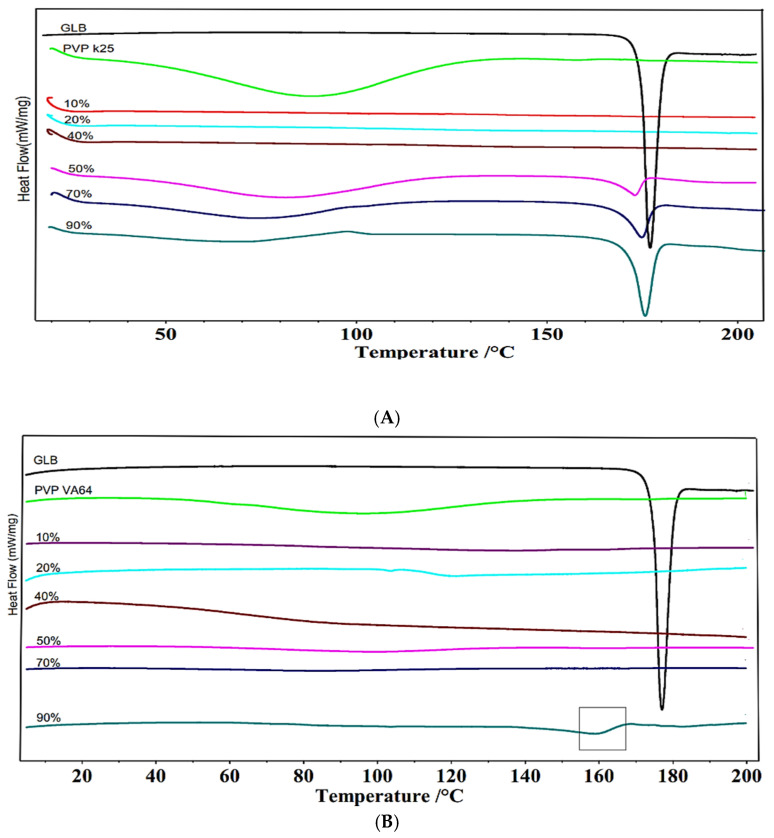

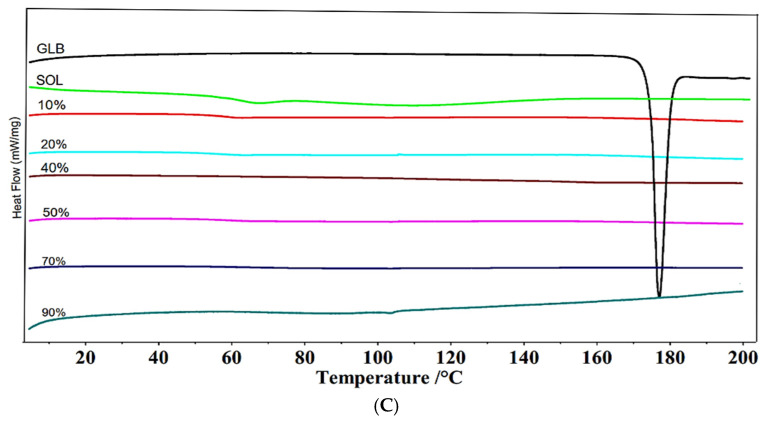
DSC thermograms of different GLB–polymer combinations. (**A**) GLB-PVP K25; (**B**) GLB-PVP VA64; and (**C**) GLB-SOL (percentages indicate drug loading).

GLB amorphization capability within each carrier was thermally assessed using DSC. The thermograms of the extrudates are depicted in Figure 1A–C. The melting point of pure crystalline GLB was established at 177 °C, consistent with previous studies [8,29,41]. For the GLB-PVP K25 system (Figure 1A), extrudates containing up to 40% GLB exhibited no discernible melting endotherm in the DSC thermograms, indicating complete amorphization of the drug within the PVP K25 matrix. However, when the GLB loading reached 50% *w*/*w*, a distinct endothermic peak emerged at approximately 174 °C, corresponding to the presence of crystalline GLB. The appearance of this melting endotherm signifies that the drug loading exceeded the miscibility limit of GLB in PVP K25, leading to phase separation and the formation of drug-rich areas capable of crystallization. The intensity of this melting peak increased progressively with higher GLB concentrations, confirming enhanced phase separation [17]. The drug loading capacity of the GLB-PVP VA64 system (Figure 1B) was found to be superior to PVP K25. The extrudates containing up to 70% *w*/*w* GLB did not show any sign of the presence of the drug crystals in their DSC thermograms. This indicates that the drug is fully amorphized and homogeneously dispersed within the polymer matrix. Only at the highest loading of 90% *w*/*w* GLB was a minor residual melting endotherm. The calculated Flory–Huggins interaction parameter (χ = 0.0015) for this system further corroborates the near-ideal miscibility between GLB and PVP VA64, explaining the notable ability of this polymer to accommodate high drug loads while maintaining a homogeneous amorphous state [20]. Interestingly, the GLB-SOL system (Figure 1C) showed full amorphization across the entire range of drug concentrations tested, from 10 to 90% *w*/*w* GLB. There was no melting endotherm corresponding to the melting of GLB in any of the DSC thermograms, suggesting that SOL possesses a great capacity to molecularly disperse GLB even at very high concentrations. The low glass transition temperature of SOL (approximately 70 °C) also facilitates molecular mobility during processing, promoting intimate mixing at the molecular level [38]. Furthermore, the amphiphilic nature of SOL also promotes molecular dispersion and inhibits drug clustering [15].

#### 3.1.3. Prediction and Validation of T_g_

**Figure 2:**
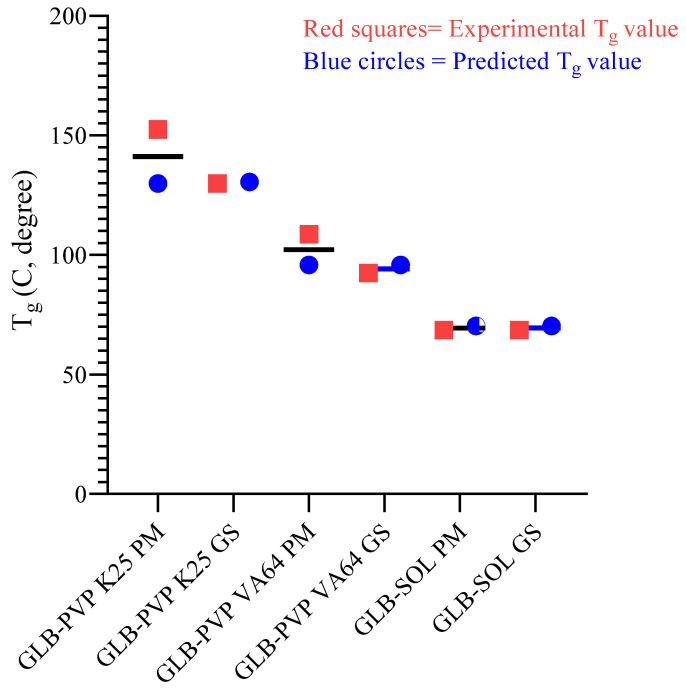
Predicted versus experimental T_g_ values for GLB–polymer loading of 10%. PM = physical mixture; GS = glassy solution.

Glass transition behaviour was further analysed to confirm phase homogeneity. One value of T_g_ that falls between the values corresponding to the amorphous drug along with the amorphous polymeric substance is evidence of uniformity on the molecular level, as would appear in a glassy solution, while the observation of dual values is evidence of phases separating into various amorphous regions. The Gordon–Taylor equation is commonly employed for estimating the value of T_g_ in binary mixtures as a theoretical standard for ideal miscibility. The consistency of the experimentally determined value of T_g_ with the value of T_g_ based on the Gordon–Taylor equation is the criterion for ideal miscibility; otherwise, the result can be attributed to particular interactions involving the drug and polymer [23,42]. The T_g_ value of GLB was found to be 63.6 °C (Figure S1) and ΔC_p_ = 0.42 J/g⋅K, consistent with previously reported values [27,43]. For this investigation, the T_g_ values of 10% *w*/*w* GLB–polymer mixtures were calculated (based on the dry mass composition) using the Gordon & Taylor equation, then compared with experimental values determined from DSC measurements of physical mixtures and hot-melt-extruded glassy solutions (Figure S2). For the 10% GLB-loaded physical mixtures, there were notable deviations between calculated and experimental values of T_g_, particularly in samples containing PVP K25 (∆T_g_ = 22.55 °C) and PVP VA64 (∆T_g_ = 12.75 °C) (Figure 2 and Table 3). Physical mixtures represent discrete domains of drug and polymer particles rather than homogeneous molecular dispersion. The T_g_ measured in physical mixtures by DSC reflects the overlapping glass transitions of the individual components; the observed values might be influenced by partial interfacial mixing during the DSC heating scan but do not indicate true molecular-level dispersion at room temperature. The deviation from the Gordon–Taylor prediction therefore simply confirms the absence of pre-existing molecular mixing in the unprocessed state. Conversely, after HMIM processing, there was excellent correspondence between predicted and experimental T_g_ values, with variations of <4 °C for the three GLB–polymer glassy solution granules. The close alignment observed for SOL-based systems (ΔT_g_ ≈ 1.75 °C for physical mixtures and 1.65 °C for extrudates) further emphasizes its inherent miscibility and efficiency in forming homogeneous dispersions. These findings clearly indicate that hot-melt extrusion effectively promotes molecular-level mixing, resulting in the formation of stable, single-phase glassy solutions. A previous study involving celecoxib-PVP has also shown similar observations, where close alignment of experimental and Gordon–Taylor model predictions of T_g_ values was attributed to successful fabrication of amorphous solid dispersions generated by HME [44]. These findings clearly indicate that the hot-melt extrusion effectively promotes molecular-level mixing, which results in the formation of a stable single-phase glassy solution.

### 3.2. Characterization of Glassy Solution Extrudates

#### 3.2.1. Extrudates Manufacturability and Clarity

**Figure 3:**
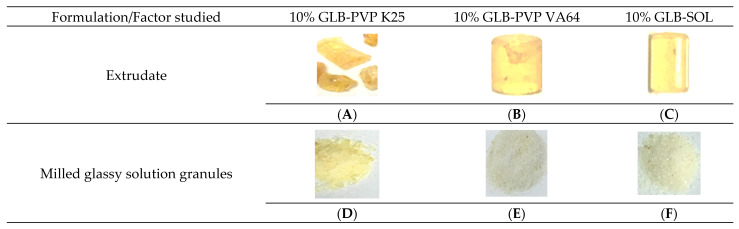
Different polymer extrudates and glassy solution granules. 10% GLB-PVP K25 extrudate (**A**); 10% GLB-PVP VA64 extrudate (**B**); 10% GLB-SOL extrudate (**C**); milled glassy solution granules from 10% GLB-PVP K25 (**D**); milled glassy solution granules from 10% GLB-PVP VA64 (**E**); milled glassy solution granules from 10% GLB-SOL (**F**).

The manufacturability of the extrudates was assessed relative to the ease of forming whole, readily retrievable strands, while the clarity was assessed as the initial indicator of glass solution formation. From Table 4, the 10% GLB compositions with PVP VA64 and SOL had the best manufacturability in terms of the production of clear and whole extrudates using a low cylinder temperature of 150 °C (Figure 3B,C). The 10% GLB-PVP K25 composition, in turn, yielded extrudates that were brittle with fissures (Figure 3A), using a high process temperature of 180 °C. The extrudate clarity was measured using a scale of one (clear) to three (unclear). A scale score of “1” meant that the extrudates were highly transparent and highly prone to glassy solution formation, and this score was assigned to all 10% GLB-PVP VA64 and 10% GLB-SOL samples. However, a score of “2” meant that those extrudates were low in clarity and prone to solid solution formation, and this score was assigned to all 10% GLB-PVP K25 samples. After milling, the extrudates were passed across a 500 µm sieve. Photographs of the obtained granules are shown in Figure 3D–F. Sieving was quick and easy for GLB-VA64 and GLB-SOL, but the process was more laborious for GLB-PVP K25. This is expected as PVP K25 has a greater glass transition temperature (~145 °C) compared to PVP-VA64 (~101 °C) and SOL (~70 °C). Characterization of the sieved granules is summarized in Table 5. For all formulations, the GLB content was within the acceptable range of 90–110%. According to the USP <1174> classification, granules sieved through 500 µm showed “Fair” flow properties that are suitable for further compression into tablets.

#### 3.2.2. XRPD

**Figure 4:**
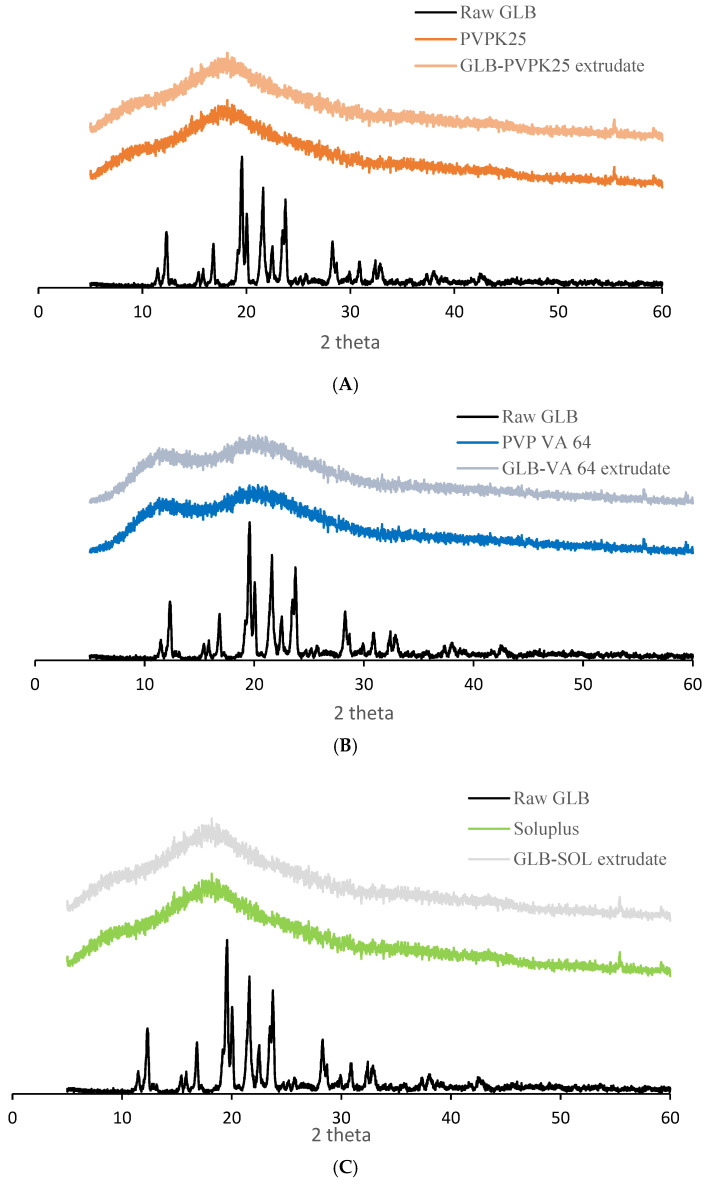
XRPD of crystalline GLB, PVP K25, PVP VA64, SOL and sieved extrudate glassy solution granules. Raw GLB, PVP K25, and GLB-PVP K25 extrudate (**A**); Raw GLB, PVP VA64, and GLB-PVP VA64 extrudate (**B**); Raw GLB, Soluplus, and GLB-SOL extrudate (**C**).

Diffractograms of unprocessed GLB (Figure 4A–C) were dominated by many sharp high-intensity diffraction peaks, demonstrating its crystallinity. The prominent peaks were noticed in the 2θ positions of 11.7°, 12.1°, 19.6°, 20.1°, 21.4°, 22.3°, 23.7°, 28.3°, 31.1°, 32.3°, and 32.5°, as was evident from its crystalline structure [29,45]. Notably, XRPD diffractograms of glassy solutions extruded with 10% (*w*/*w*) drug load (GLB-PVP K25, GLB-PVP VA64, and GLB-SOL) showed no difference from XRPD diffractograms of their pure polymers. The absence of Bragg reflections of crystalline GLB indicates a loss of long-range order of its molecules and their solubilization at a molecular level in amorphous polymers. However, it is important to acknowledge the detection limitations of XRPD for quantifying low levels of crystallinity in amorphous solid dispersions [46]. Overall, this XRPD finding provides additional structural validation for amorphization that aligns perfectly with the thermal and miscibility data. It is important to acknowledge the detection limitations of XRPD for quantifying low levels of crystallinity in amorphous solid dispersions.

#### 3.2.3. FTIR

**Figure 5:**
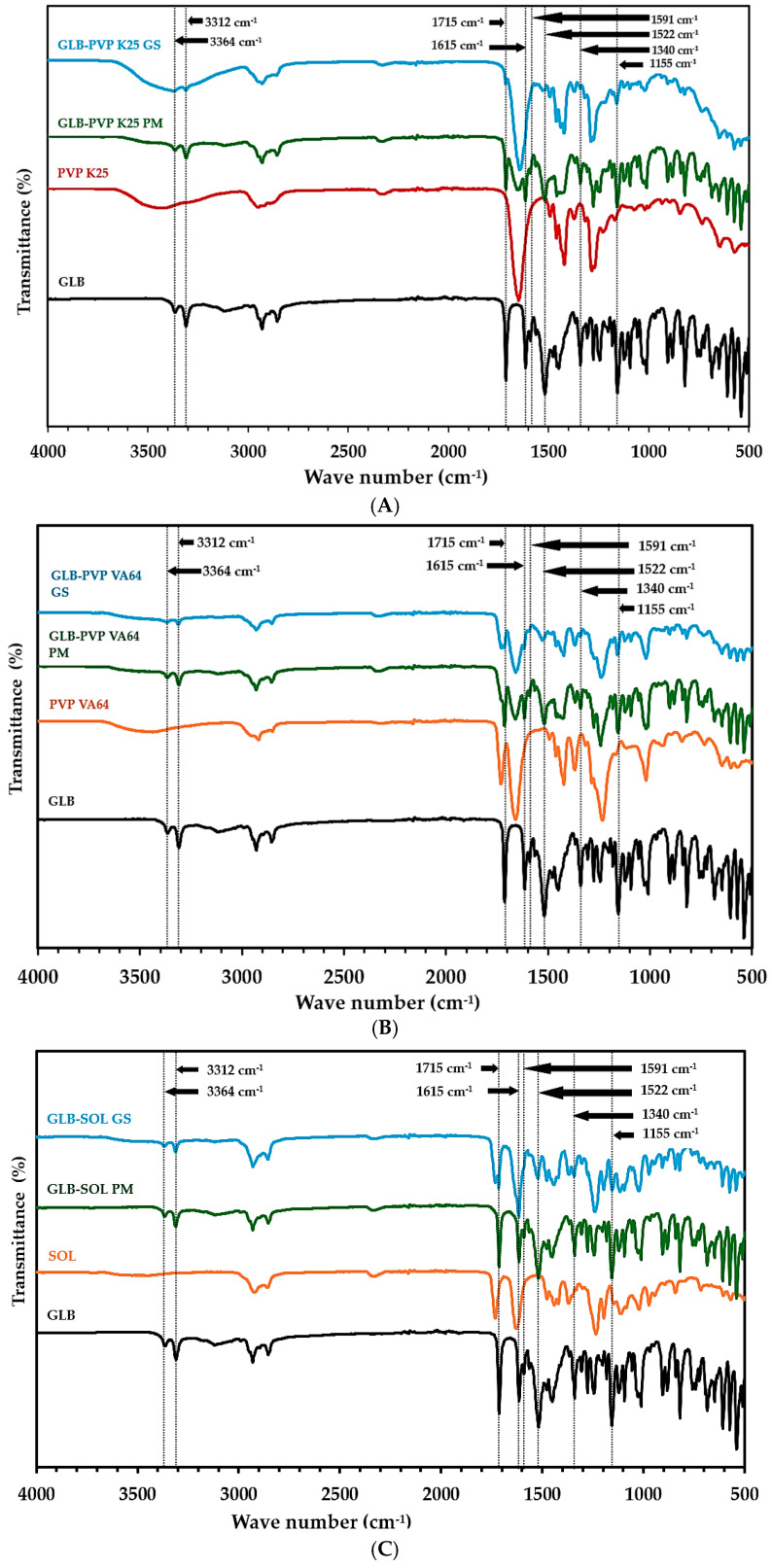
Powder FT-IR spectra for GLB, PVP K25, PVP VA64, SOL, their physical mixtures (PMs) and glassy solution granules (GSs). GLB, PVP K25, GLB-PVP K25 PM and GLB-PVP K25 GS (**A**); Raw GLB, PVP VA64, GLB-PVP VA64 PM and GLB-PVP VA64 GS (**B**); Raw GLB, Soluplus, GLB-SOL PM and GLB-SOL GS (**C**).

Vibrational spectroscopy, such as FTIR, is frequently used to characterize amorphous solid dispersions by identifying intermolecular drug–polymer interactions [47]. The absence, intensity reduction, or shifting of characteristic vibrational bands in the spectrum of a drug–polymer system is widely accepted as evidence of molecular interactions, such as hydrogen bonding or dipole–dipole interactions [48]. Hydrogen bond formation, X–H···A (where A is the acceptor atom), also results in a broader peak, whereas the unbonded X–H stretch generates sharp peaks [49]. The FTIR spectrum of unprocessed GLB (Figure 5) displayed characteristic peaks at 3312 and 3364 cm^−1^ (N–H stretch), 1715 cm^−1^ (urea C=O stretch), and 1522 cm^−1^ (N–H bend), C=C stretching bands at 1591 and 1519 cm^−1^, and symmetric and asymmetric S=O stretching at 1340 and 1159 cm^−1^. These values were consistent with established literature [50,51]. Consistent with a previous study [52], PVP K25 revealed a strong FTIR peak at 1650 cm^−1^ due to the C=O stretching vibration, and a broad strong peak at 3440 cm^−1^ due to the O–H stretching vibration (Figure 5A). However, the FTIR spectrum of PVP VA64 differed from that of PVP K25, showing two additional peaks at 1732 cm^−1^ (ester C=O stretching) and 1239 cm^−1^ (C–O stretching), in addition to the known peaks at 1671 cm^−1^ (amide C=O stretching) and 1285 cm^−1^ (C–N stretching) [53] (Figure 5B). SOL showed peaks at 3460 cm^−1^ for O–H stretching and at 2920 and 2856 cm^−1^ for C–H aliphatic stretching, as well as carbonyl stretching vibrations at 1736 and 1627 cm^−1^ for the ester and amide groups, respectively. Previous researchers reported similar observations [54,55] (Figure 5C). The FTIR results of GLB–polymer physical mixtures (PMs) appeared to be a summation of drug and polymer spectra (Table 6). All characteristic GLB bands were preserved with no significant shifts, although some bands showed reduced intensities due to polymer dilution, suggesting the absence of interactions between components. All characteristic GLB bands were preserved for GLB–polymer physical mixtures (PMs) (Table 6), with no shifts, although some bands showed reduced intensities due to polymer dilution, suggesting the absence of interactions between components [49]. Conversely, the glassy solutions (GSs) exhibited several spectral changes. The FTIR spectrum of GLB-PVP K25 GS showed marked broadening and reduction in intensity of the N–H stretching bands (3312, 3364 cm^−1^), the urea C=O stretch (1715 cm^−1^), the C=C stretching bands (1591 and 1519 cm^−1^), and the symmetric and asymmetric S=O stretching bands (1340 and 1159 cm^−1^) when compared to the similarly constructed physical mixture (Figure 5 and Table 6). Similar observations were made in the spectrum of GLB-PVP VA64 GS, where the N–H stretching bands at 3312 and 3364 cm^−1^ were reduced and broadened, and the urea C=O stretch at 1715 cm^−1^ was significantly reduced in intensity. Regarding GLB-SOL GS, the N–H stretches at 3312 and 3364 cm^−1^ were markedly reduced; the C=C stretch at 1591 cm^−1^ was almost entirely absent. This loss of distinctive vibration features directly supports the disruption of the long-range lattice ordering of GLB and its molecular dispersion at the microscopic level within the polymer matrix [56], consistent with the peak broadening typically observed in amorphous solids due to altered hydrogen-bonding networks [57].

#### 3.2.4. SEM

**Figure 6:**
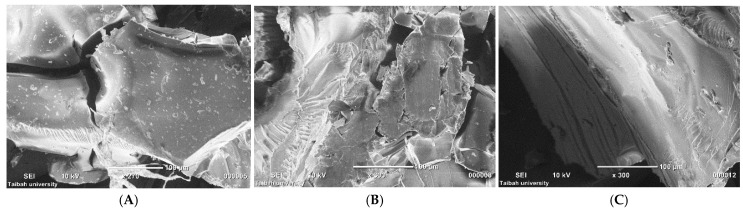
SEM micrographs of milled extrudate glassy solution granules. GLB-PVP K25, 270× magnification (**A**), GLB-PVP VA64, 300× magnification (**B**) and GLB-SOL, 300× magnification (**C**).

Morphology of the milled extrudate granules was explored via SEM (Figure 6A–C). As illustrated in Figure 6A, the GLB-PVP K25 granules showed surface particles with irregular surfaces, suggesting incomplete solubilization of GLB within the PVP K25 matrix and poor molecular-level dispersion. By contrast, both GLB-PVP VA64 (Figure 6B) and GLB-SOL (Figure 6C) granules were smooth and homogeneous with no particulate matter present. The non-existence of any visible drug crystals or phase-separated domains indicates that PVP VA64 and SOL were effective in the molecular-level solubilization of GLB, supporting the successful preparation of homogeneous glassy solutions.

### 3.3. Characterization of Tablets Containing Glassy Solution Granules

The physicochemical properties of the prepared GLB tablets comprising polymer extrudates were determined (Table 7). All tablets exhibited excellent weight uniformity as determined by their average weight being 200 mg. The tablets met the pharmacopeial requirements for friability (<0.5%) and hardness (Kp 4–6), indicating good mechanical performance. Disintegration was rapid for all formulations but varied; GLB-K25-tab disintegrated fastest at 11 s, followed by GLB-SOL-tab (20 s) and GLB-PVP VA64-tab (25 s). This rank order inversely correlates with the observed tablet hardness, suggesting that the intense compaction of the VA64 batch moderately delayed breakdown. GLB content of all formulations ranged between 97.5% and 98.3% of the theoretical drug dose. This designates a precise and uniform GLB distribution in the tablets produced by direct compression. Generally, findings here confirmed that the tablets studied exhibited acceptable properties, and none of the three polymers showed significant differentiation from the others with respect to hardness content and disintegration.

### 3.4. In Vitro Dissolution Testing

**Figure 7:**
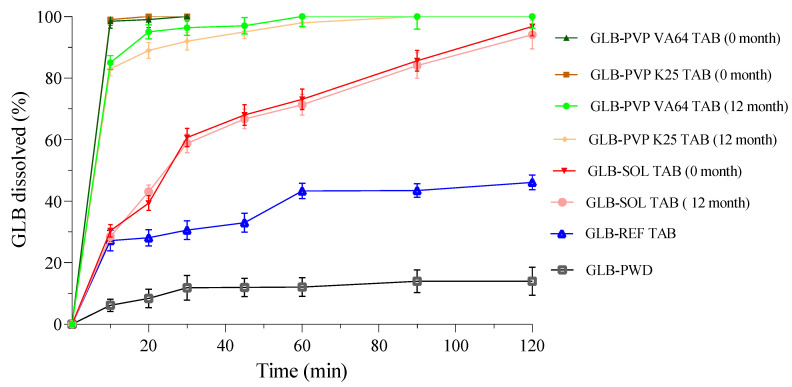
Dissolution rate profile for GLB powder, reference product and tablet formulations loaded with glassy solution granules.

GLB powder (GLB-PWD), GLB glassy solution tablet formulations GLB-K25 tab, GLB-VA64 tab, and GLB-SOL tab, and reference product (GLB-REF) dissolution profiles are presented in Figure 7. This comparative dissolution profile study was performed in a phosphate buffer (pH 6.8) as the discriminatory dissolution medium [30]. The unprocessed drug particles showed very slow dissolution, reaching only 14% dissolved after 2 h, a direct consequence of its limited aqueous solubility and great hydrophobicity, ranking GLB with the BCS Class II medications [8]. The commercial reference product (GLB-REF) showed a moderately improved dissolution profile of approximately 48% after 2 h. This is plausibly a result of using a micronized GLB, a recognized strategy to enhance surface area and dissolution rate for such drugs. Alternatively, GLB glassy solution formulations GLB-K25 tab, GLB-VA64 tab, and GLB-SOL tab revealed a superior drug release, achieving near-complete dissolution (~100%) within the 120-min study. All test formulations (K25, VA64 and SOL) produced *f*_2_ values significantly below 50 (11.1, 11.2 and 38.4, respectively). This confirms that the developed formulations are not “similar” to the reference because they provide considerably superior dissolution rates. Such a profound enhancement is rationally accredited to formulating GLB as an amorphous solid dispersion (glass solution) within the polymeric matrices. In this amorphous, molecularly dispersed state, GLB rapidly generates supersaturation upon dissolution. Despite the similarly rapid disintegration times (less than 1 min) for these tablets, their dissolution kinetics differed markedly, highlighting the critical influence of polymer choice. Tablets fabricated from PVP K25 and PVP VA64 extrudates displayed an immediate dissolution, achieving ~100% within 10 min. The rapid release of GLB for these formulations can be explained in terms of the physicochemical properties of the polymeric carriers, in addition to the amorphous nature of the drug. PVP K25 and PVP VA64 are highly hydrophilic polymers, driven by the polar lactam (N-vinyl-2-pyrrolidone) groups in their structure, which facilitate instant water uptake, thus promoting hydration and dissolution of the matrices [58]. Upon swift polymer dissolution, the amorphous GLB will be liberated into the medium with minimal impediment, allowing for instant supersaturation generation, consistent with their hydrophilic characteristic and relatively low solution viscosity [12]. Conversely, GLB-SOL-tab presented a marked biphasic release feature with an initial fast phase of ~60% dissolution in 20 min, followed by a prolonged slower phase up to completion. The biphasic release behaviour observed for the SOL-based formulations could be a result of the unique amphiphilic graft copolymer structure. SOL consists of polyvinyl caprolactam and polyvinyl acetate segments, which are relatively hydrophobic, grafted onto a hydrophilic polyethylene glycol (PEG) backbone [15]. Upon contact with the dissolution media, the hydrophilic PEG chains promote rapid water uptake and swelling, resulting in an initial burst release of GLB molecules located near the surface of tablets. Concurrently, the amphiphilicity of SOL facilitates self-assembly with the generation of polymeric micelles that enhance drug solubilization and help maintain the state of supersaturation in dissolution media. As dissolution progresses, the hydrophobic domains of the SOL matrix contribute to a diffusion-controlled release of GLB, leading to a second slower release phase [15,59,60]. Overall, while all glassy solution formulations successfully converted the crystalline GLB into a readily dissolving amorphous form, exceeding both the unprocessed drug and the commercial reference tablets, the release profile was finely tuned by the polymeric carrier. PVP K25 and PVP VA 64 facilitated immediate and complete dissolution with suitability for quick absorption. SOL, however, presented a more controlled, biphasic release, which could be advantageous for prolonging supersaturation or mitigating potential precipitation.

### 3.5. Dissolution Kinetic Analysis

As observed in the dissolution section, the PVP-based tablets (VA64 and K25) exhibited immediate-release characteristics, achieving complete dissolution (99%) within 10 min. The first order model provided the best fit (R^2^ = 0.995 and 0.998 for GLB-PVP VA64 and GLB-PVP K25, respectively), confirming that the release rate is principally a function of the concentration gradient between the stagnant layer and the bulk medium (Table 8). However, the Higuchi model demonstrated superior correlation (R^2^ = 0.991) for GLB-SOL TAB, indicating that drug release is governed by Fickian diffusion from a matrix system.

### 3.6. Stability Testing

**Figure 8:**
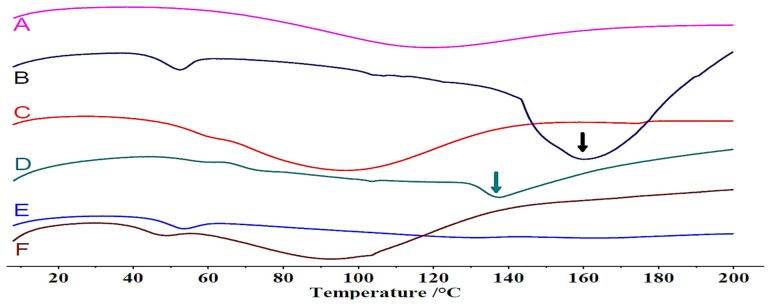
Thermal analysis of 10% GLB–polymers glassy solutions (GSs). GLB-PVP K25 at 0 M (**A**), GLB-PVP K25 at 12 M (**B**), GLB-PVP VA64 at 0 M (**C**), GLB-PVP VA64 at 12 M (**D**), GLB-SOL at 0 M (**E**) and GLB-SOL at 12 M (**F**).

The stability of an amorphous solid dispersion is critical in developing successful products for commercialization, as recrystallization might counteract solubility benefits and make oral bioavailability irregular [61]. In the 12-month stability testing, the results for the three polymeric matrices disclosed key differences in the effectiveness of the matrices in preventing recrystallization of GLB in the amorphous state. The physical state transitions occurring within the granules were confirmed through the application of DSC (Figure 8). For the glassy solution of GLB-PVP K25, after the completion of 12 months, the DSC thermogram displayed a melting endotherm at approximately 165 °C, characteristic of recrystallized GLB (Figure 8B). This confirmed the inadequacy of PVP K25 in stabilizing GLB for this period at this concentration level [23]. Contrastingly, the GLB-PVP VA64 glassy solutions exhibited a better stability profile. The DSC scan of the glassy solution of GLB-PVPVA 64 after 12 months revealed only a small endotherm near 140 °C, possibly reflecting limited amorphous–amorphous phase separation (Figure 8D). This correlates well with the established anti-plasticizing effects of PVP-based copolymers that prevent crystallization at the molecular level [62]. In turn, the GLB-SOL blend showed excellent stability, with no observed recrystallization in the period of 12 months (Figure 8F). The large endotherm within 50–110 °C was related to the uptake of water by the SOL polymer, an expected feature of its hygroscopic character, with no adverse influence on the stability of the resultant amorphous dispersion [63]. The dissolution performance of tablets made from aged granules was a direct reflection of the physical stability results shown in Figure 7. Tablets containing GLB-PVP VA64 and GLB-SOL granules retained their superior dissolution profiles with no statistically significant reduction in GLB dissolution patterns compared to fresh tablets at T_0_, confirming that the stabilizing interactions were preserved within the glassy solution. On the other hand, the results obtained using the GLB-PVP K25 tab showed a decrease in GLB dissolution, with over 10% less drug released at the early time points. The decreased dissolution rate is a direct consequence of the physical instability (recrystallization) revealed by the DSC analysis, as crystalline GLB restores the intrinsic dissolution limitations of its raw form. The formation of single-phase homogeneous glassy solutions in PVP VA64 and SOL systems provided an effective molecular constraint against recrystallization. The potential for recrystallization beyond this 12-month period was not evaluated in this study. The *f*_2_ value for GLB-SOL-tab was 73, which substantially exceeds the critical value of 50, confirming similarity between the initial and aged dissolution profiles. With more than 85% dissolution within 15 min, the *f*_2_ value is not necessary for GLB-PVP VA64 and GLB-PVP K25 and dissolution profiles are accepted as similar without further mathematical evaluation.

### 3.7. Bioavailability

**Figure 9:**
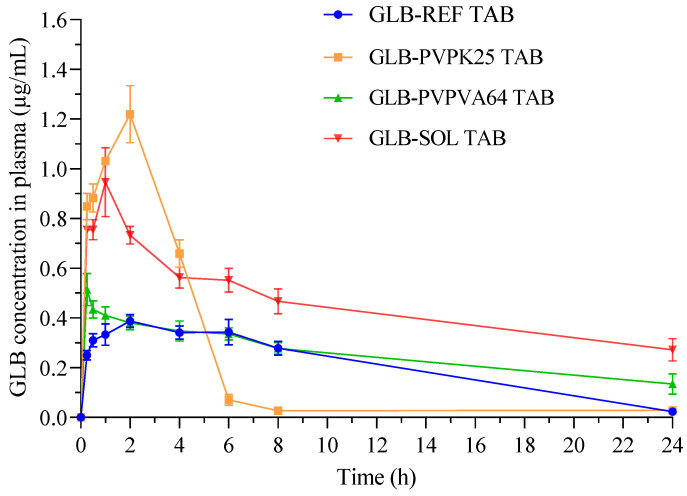
Plasma concentration–time curves for reference glibenclamide product and tested tablets containing glassy solution polymer extrudates.

The plasma concertation–time profiles and the key pharmacokinetic parameters for GLB tablets orally administered to rats are shown in Figure 9 and Table 9, respectively. The C_max_ values for glassy solution formulations GLB-PVPK25 TAB, GLB-PVPVA64 TAB and GLB–SOL TAB were 1.22 ± 0.12, 0.51 ± 0.07 and 0.95 ± 0.14 µg/mL, respectively, all of which were significantly greater (*p* < 0.05) than that of GLB-REF TAB (0.39 ± 0.10 µg/mL). The T_max_ value was 2 h for both of GLB–REF TAB and GLB-PVPK25 TAB; however, it was reached at 1 h and 0.25 h for GLB-SOL TAB and GLB-PVPVA64 TAB, respectively. The values of C_max_ and T_max_ revealed that GLB was quickly absorbed into the bloodstream after being rapidly dissolved in the gastrointestinal tract (GIT). The respective AUC_0–24h_ values of GLB–REF TAB, GLB–PVPK25 TAB, GLB–PVPVA64 TAB and GLB-SOL TAB were 4.58 ± 0.62, 4.33 ± 0.41, 5.69 ± 0.64 and 11.28 ± 0.64 µg·h/mL. Although GLB-PVP K25 showed the highest C_max_, its overall exposure (AUC_0–24h_) was not significantly improved relative to the reference, which indicated limited sustained absorption. The comparatively low bioavailability of GLB-PVP K25 formulation, despite its prompt dissolution, can be attributed to inability of the polymer to sustain drug supersaturation in vivo. PVP K25 is a very hydrophilic polymer that dissolves quickly, creating a supersaturated drug solution [64]. However, due to its low molecular weight, lack of amphiphilicity and dilution in the complex GIT environment, PVP K25 provides inadequate stability against drug precipitation [65,66]. Research conducted on other BCS Class II drugs has indicated that lower molecular weight PVP may offer inadequate steric hindrance or “crystallization inhibition” compared to higher molecular weight PVP, thus increasing the precipitation rates of the drugs in the body, consequently reducing the AUC [67]. The matching calculated values of relative bioavailability (F%) of the previous formulations were 100.00, 94.50, 124.5 and 246.30%, confirming significant enhancement of GLB bioavailability in both formulations that showed successful glassy solution formation: GLB-PVPVA64 and GLB-SOL (*p* < 0.05). The greater enhancement in relative bioavailability for SOL-containing formulation might be attributed to the reported ability of this polymer to act as a solubilizer and inhibitor of GLB precipitation with the formation of GLB protective micelles [68,69]. The amphiphilic characteristics of SOL and its low critical micelle concentration facilitate the formation of GLB containing micelles that have high ability to resist GIT pH changes and dilution. Collectively, the in vivo results support the ability of glassy solution-based tablets in bioavailability enhancement of GLB. Future research using diabetic animal models or clinical trials in human patients would be valuable to validate these findings under pathophysiologically relevant conditions.

## 4. Conclusions

This research confirms the feasibility of the hot-melt injection molding (HMIM) method for the preparation of glibenclamide glassy solutions. Hansen solubility parameters, Flory–Huggins interaction parameters, and Gordon–Taylor equations successfully predicted GLB miscibility behaviour relative to the tested polymers. In turn, agreement was found with the experimentally detected high compatibility and stabilizing properties of the carriers. Of these, PVP VA64 and SOL were found to be more compatible and stabilizing than PVP K25. HMIM processing helped in achieving complete amorphization of GLB, which formed tablets with strong mechanical strength and remarkably better dissolution characteristics. The long-term stability analysis revealed that GLB existed in amorphous form for 12 months in PVP VA64/SOL matrices, whereas partial crystallization took place in PVP K25. The physicochemical improvements contributed to remarkably higher bioavailability. The findings demonstrated in this work establish HMIM as a robust platform for enhancing the oral performance of dissolution-limited antidiabetic agents.

## Figures and Tables

**Table 1:** Solid solution loaded tablet formulations.

Ingredients	GLB-K25-tab (mg %)	GLB-VA64-tab (mg %)	GLB-SOL-tab (mg %)
GLB-K25-GSG	12.5	-	-
GLB-VA64-GSG	-	12.5	-
GLB-SOL-GSG	-	-	12.5
Microcrystalline cellulose	81.8	81.8	81.8
Colloidal silicon dioxide	0.2	0.2	0.2
Crospovidone	5	5	5
Magnesium stearate	0.5	0.5	0.5

**Table 2:** Hansen solubility parameter differences and predicted Flory–Huggins interaction parameters (χ) for glibenclamide–polymer systems.

System	True Density (g·cm^−3^)	Δδ (MPa^1/2^)	χ	Predicted Miscibility
GLB	1.37	–	–	–
PVP K25	1.18	1.6	0.373	Miscible
SOL	0.99	1.7	0.421	Miscible
PVP VA64	1.167	0.1	0.0015	Miscible

**Table 3:** Predicted and experimental Tg values of GLB–polymer systems.

System	Predicted T_g_ (°C)	Experimental T_g_ (Physical Mixture, °C)	Experimental T_g_ (Glassy Solution, °C)	ΔT_g_ (Physical Mixture)	ΔT_g_ (Glassy Solution)
GLB-PVP K25	129.90	152.45	130.50	+22.55	+0.60
GLB-PVP VA64	95.85	108.60	92.45	+12.75	−3.40
GLB-SOL	70.25	68.50	68.60	−1.75	−1.65

**Table 4:** Extrudates clarity and manufacturability.

Formulation/Factor Studied	10% GLB-PVP K25	10% GLB-PVP VA64	10% GLB-SOL
Clarity	2	1	1
Manufacturability	Difficult No physically intact clear extrudates were obtained Temperature higher than drug melting point is required for injection molding	Easy Physically clear intact extrudates were obtained Temperature lower than drug melting point is required for injection molding	Easy Physically clear intact extrudates were obtained Temperature lower than drug melting point is required for injection molding
Sieving process	-Takes more time and only part of the extrudates pass through due to extrudate hardness	-Fast and easy sieving process and all extrudates were size reduced through sieve	-Fast and easy sieving process and all extrudates were size reduced through sieve

**Table 5:** Characterization of sieved glassy solutions.

Characterization	GLB-PVP K25	GLB-PVP VA64	GLB-SOL
Bulk density (mg/mL)	0.45	0.48	0.49
Tapped density (mg/mL)	0.55	0.59	0.6
Carr’s index (%)	18.1	18.9	18.3
GLB content (%)	98.7	98.2	99.2

**Table 6:** FTIR peak assignments and positions for GLB and changes in GLB–polymer combinations.

Assignment	GLB (cm^−1^)	GLB-PVP K25 PM (cm^−1^)	GLB-PVP K25 GS (cm^−1^)	GLB-PVP VA64 PM (cm^−1^)	GLB-PVP VA64 GS (cm^−1^)	GLB-SOL PM (cm^−1^)	GLB-SOL GS (cm^−1^)
N–H stretch (secondary amine)	3364	3364	3365 (broadened, intensity ↓)	3364	3364 (broadened, reduced intensity)	3364	3364 (broadened, intensity ↓)
N–H stretch (urea)	3312	3312	3308 (broadened, intensity ↓, shifted: −4 cm^−1^)	3312	3310 (broadened, intensity ↓, shifted: −2 cm^−1^)	3312	3309 (broadened, intensity ↓, shifted: −3 cm^−1^)
Urea C=O stretch	1715	1715	1715 (near disappearance)	1715	1715 (broadened, intensity ↓)	1715	1715 (broadened)
Aromatic C=C	1615	1615	1615 (broadened, intensity ↓)	1615	1615 (broadened, intensity ↓)	1615	1615 (broadened, intensity ↓)
Aromatic ring vibration	1591	1591	1590 (near disappearance)	1591	−(disappearance)	1591	−(disappearance)
N–H bend (amide II)	1522	1522	1522 (broadened, intensity ↓)	1522	1522 (broadened, intensity ↓)	1522	1522 (broadened, intensity ↓)
C–N stretch	1340	1340	1340 (broadened, intensity ↓)	1340	1340 (broadened, intensity ↓)	1340	1340 (broadened, intensity ↓)
S=O stretch (sulfonylurea)	1155	1155	1155 (broadened, intensity ↓)	1155	1155 (broadened, intensity ↓)	1155	1155 (broadened, intensity ↓)

PM: physical mixture; GS: glassy solution; ↓ indicates decrease in intensity.

**Table 7:** Characterization of tablets containing glassy solutions.

Characterization	GLB-PVP K25-tab	GLB-PVP VA64-tab	GLB-SOL-tab
Tablet weight (mg)	199.2 ± 5	201.4 ± 8	200 ± 7
Hardness (Kp)	4 ± 0.2	5 ± 0.1	4.5 ± 0.1
Friability (%)	0.44	0.45	0.46
Disintegration (s)	11 ± 0.1	25 ± 0.3	20 ± 0.2
Content of GLB per tablet (%)	98	97.5	98.3

**Table 8:** Dissolution kinetic modelling for GLB formulations.

Formulation	Zero-Order (R^2^)	First-Order (R^2^)	Higuchi (R^2^)	Hixson-Crowell (R^2^)
GLB-REF TAB	0.902	0.941	0.982	0.925
GLB-PVP VA64	0.814	0.995	0.921	0.884
GLB-SOL TAB	0.958	0.972	0.991	0.965
GLB-PVP K25	0.795	0.998	0.905	0.862

**Table 9:** Pharmacokinetic profiling of tested GLB tablets.

Parameters	GLB-PVPK25 TAB	GLB-PVPVA64 TAB	GLB-SOL TAB	GLB-REF TAB
C_max_ (µg/mL)	1.22 ± 0.12	0.51 ± 0.07	0.95 ± 0.14	0.39 ± 0.10
T_max_ (h)	2.0 ± 0.00	0.25 ± 0.00	1.0 ± 0.00	2.0 ± 0.00
AUC _0–24h_ (µg·h/mL)	4.33 ± 0.52	5.69 ± 0.46	11.28 ± 0.64	4.58 ± 0.62
F%	94.5	124.5	246.3	100

## Data Availability

The original contributions presented in this study are included in the article. Further inquiries can be directed to the corresponding author.

## Supplementary Materials

The following supporting information can be downloaded at: https://www.mdpi.com/article/10.3390/pharmaceutics18040421/s1, Figure S1: T_g_ determination of pure glibenclamide; Figure S2 T_g_ determination of 10% GLB combined with different polymers; PVP K25, PVP VA64 and SOL.
