# Supplementary material for: Hot-Melt Processed Glibenclamide Glassy Solutions: A Novel Oral Delivery Platform for Enhanced Bioavailability in Diabetes
Source: Pharmaceutics. 2026 Mar 30;18(4):421. doi: 10.3390/pharmaceutics18040421 (PMC13118826; doi:10.3390/pharmaceutics18040421)
Supplement: Supplementary file 1 [file pharmaceutics-18-00421-s001.zip › pharmaceutics-4185823-supplementary.pdf]

Figure supplements

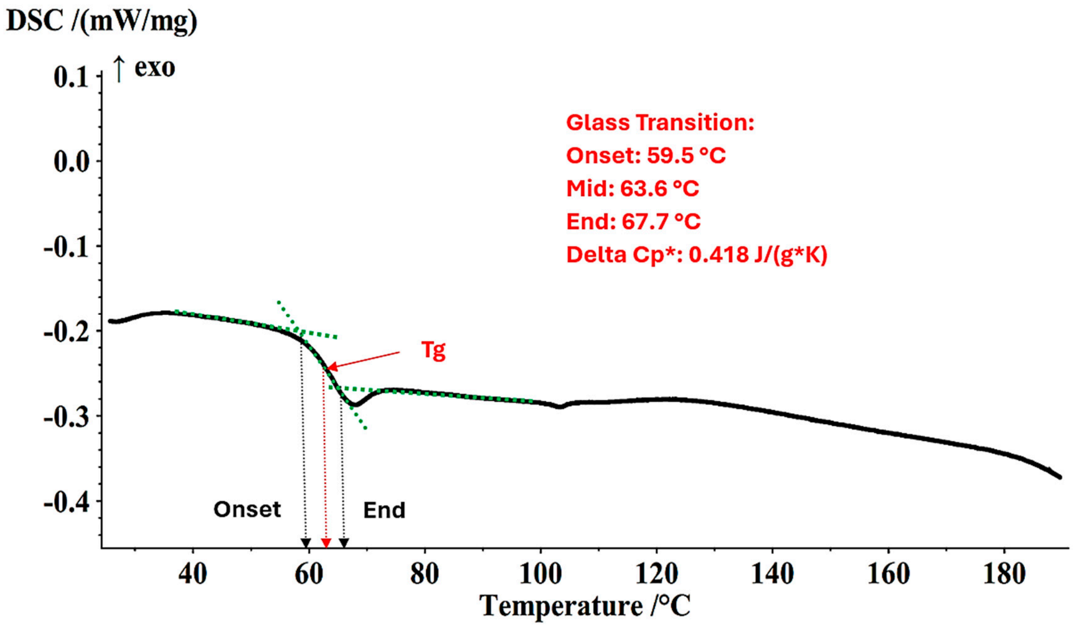

Figure S1: T_g determination of pure glibenclamide

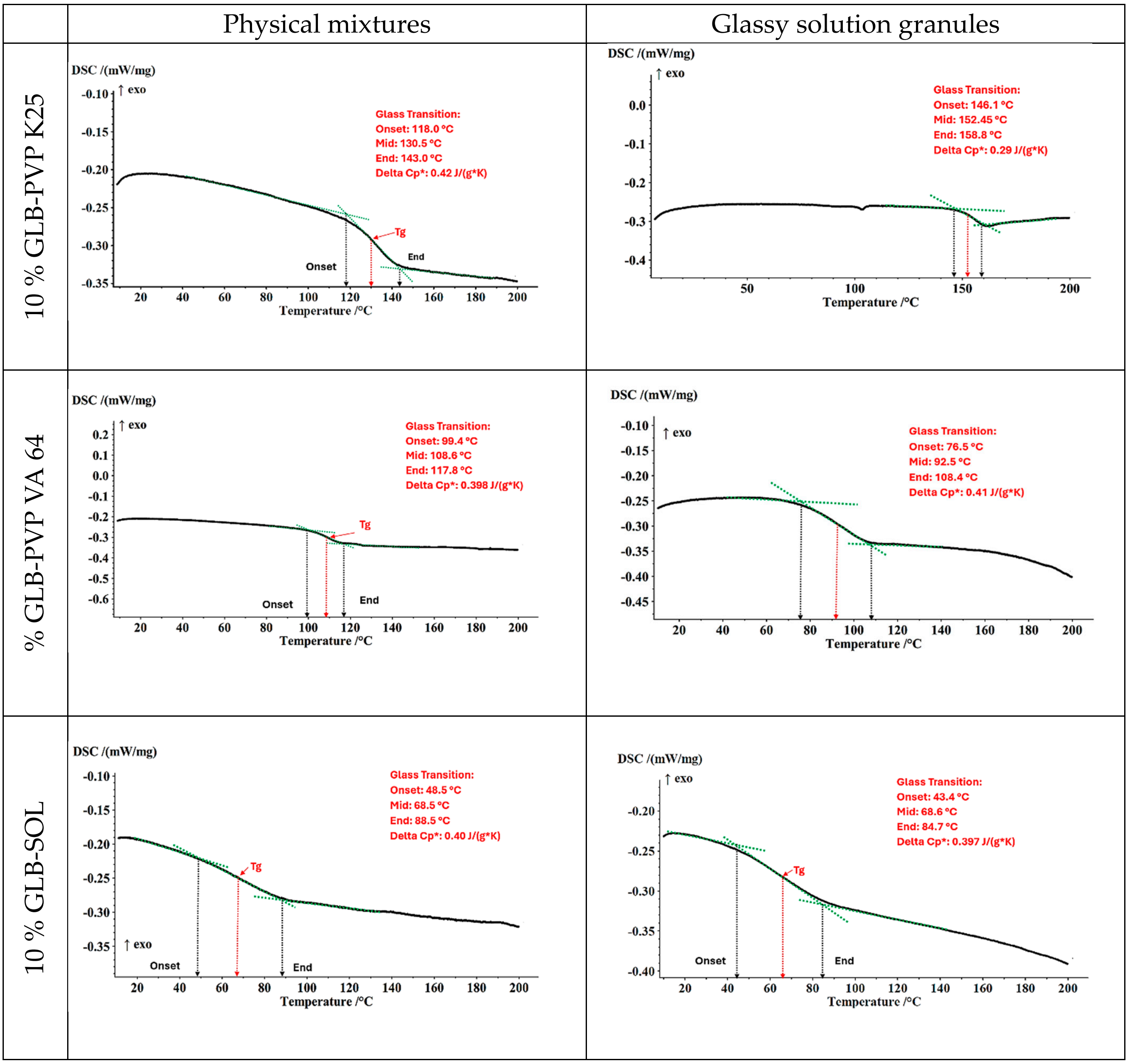

Figure S2: T_g determination of 10% GLB combined with different polymers; PVP K25, PVP VA64 and SOL.
